# The Relationship between Selected Bioelements and Depressiveness Associated with Testosterone Deficiency Syndrome in Aging Men

**DOI:** 10.3390/medicina56030125

**Published:** 2020-03-13

**Authors:** Iwona Rotter, Adrian Wiatrak, Aleksandra Rył, Katarzyna Kotfis, Żaneta Ciosek, Maria Laszczyńska, Olimpia Sipak-Szmigiel, Aleksandra Szylińska

**Affiliations:** 1 Department of Medical Rehabilitation and Clinical Physiotherapy, Pomeranian Medical University, 71-210 Szczecin, Poland; iwrot@wp.pl (I.R.); wiaadr@gmail.com (A.W.); ciosekzaneta@gmail.com (Ż.C.); aleksandra.szylinska@gmail.com (A.S.); 2 Department of Anesthesiology, Intensive Therapy and Acute Intoxications, Pomeranian Medical University, 71-210 Szczecin, Poland; katarzyna.kotfis@pum.edu.pl; 3 Department of Histology and Developmental Biology, Pomeranian Medical University in Szczecin, 71-210 Szczecin, Poland; laszcz@pum.edu.pl; 4 Department of Obstetrics and Pathology of Pregnancy, Pomeranian Medical University in Szczecin, 71-210 Szczecin, Poland; olimpiasipak-szmigiel@wp.pl

**Keywords:** testosterone deficiency syndrome, depression, testosterone, bioelements

## Abstract

*Background and Objectives:* Abnormal concentrations of bioelements (magnesium, manganese, chromium, copper, zinc) have been associated with physical and emotional dysfunctions, including depression. This association, however, has not been analyzed in testosterone deficiency syndrome (TDS) or patients with depressiveness, i.e., when individual symptoms do not form the picture of a full-syndrome depressive disorder. This study aimed to assess the relationship between concentrations of selected bioelements and the incidence of depressive symptoms in men aged 50 years and older with a concurrent testosterone deficiency syndrome. *Material and Methods:* Blood samples were taken from 314 men; the mean age of the population was 61.36 ± 6.38 years. Spectrophotometric method for biochemical analysis of magnesium (Mg), manganese (Mn), chromium (Cr), copper (Cu), and zinc (Zn) was used. The diagnosis of testosterone deficiency syndrome (TDS) was based on the total testosterone (TT), free testosterone (FT), estradiol (E2), and dehydroepiandrosterone sulfate (DHEAS) levels by ELISA. Each participant completed the Androgen Deficiency in Aging Male (ADAM) questionnaire, as well as the Beck Depression Inventory (BDI-Ia) measuring the severity of depressive symptoms. *Results:* Emotional disturbances manifested as depressive symptoms were diagnosed in 28.7% of all participants and testosterone deficiency syndrome in 49.3%. In the TDS group, the analysis showed a significant correlation between the level of manganese (*R* = 0.225, *p* = 0.005) and chromium (*R* = 0.185, *p* = 0.021) with the incidence of depression. *Conclusions:* The results of our study demonstrated a relationship between manganese and chromium concentrations with the incidence of depression in men aged 50 years and older with a concurrent testosterone deficiency syndrome. This may indicate that there is a correlation between these bioelements, as well as emotional disorders manifested as depressive symptoms in aging men with a diagnosed testosterone deficiency.

## 1. Introduction

It is estimated that the prevalence of depression and depressiveness, i.e., individual symptoms that do not form the picture of a full-syndrome depressive disorder, increases among people over 65 years old [1]. The most common symptoms are mood deterioration, a decrease in motivation and energy levels, negative self-perception, anxiety, concentration problems, difficulties maintaining an erection, loss of appetite, and decrease in physical activity [2,3]. 

The reasons for the occurrence of depressive changes can be divided into psychosocial factors, related to loss of employment, financial independence, as well as change in social position, and into biological factors, among which the most important are degeneration of the central nervous system progressing with age and a reduction of neurotransmitter activity [3,4].

Clinical depression and single depressive symptoms in aging men may be caused by a gradual decrease in testosterone concentration associated with aging, which may eventually lead to testosterone deficiency syndrome (TDS) [5,6]. The most important symptoms of this syndrome are erectile dysfunction, decreased libido, gynecomastia, decreased muscle strength, testicular volume, and overall body weight. Often these symptoms are also accompanied by changes at the emotional level, which are also characteristic of the occurrence of depression. These include sleep and concentration disorders, irritability, and a decrease in motivation [7,8].

The relationship between the incidence of TDS and depressive disorders may be associated with the neuroprotective effect of testosterone achieved by inhibiting the toxic effect of glutamate [9,10].

The glutamate activates the α-amino-3-hydroxy-5-methyl-4-isoxazolepropionic acid receptor (AMPA) and *N*-methyl-D-aspartate receptor (NMDA), which results in increased Ca^2+^ transport [11]. This activates endonucleases and proteases enzymes, which damage elements of the cytoskeleton, cell membranes, and nerve cell DNA. In addition, the hormone also inhibits the 5-hydroxytryptamine 3 (5-HT3) receptor, which is responsible for the serotonin uptake in the synaptic cleft, thereby reducing the stimulation of the target cell [12]. Antidepressants from the selective serotonin reuptake inhibitor (SSRIs) group have a similar effect.

When analyzing the pathophysiology of depression symptoms, we should note the changes in concentrations of bioelements, as they may underlie the mechanisms leading to emotional disorders as well as clinical depression [13,14,15,16]. In suffering patients, magnesium supplementation has been used, as it affects several biochemical pathways in the brain responsible for apathy [17]. Zinc and copper, like testosterone, modulate the glutamatergic system by inhibiting NMDA receptor activity [18,19]. Moreover, the increase in manganese concentration, which can be observed in employees of the metallurgical and chemical industries, can exhibit neurotoxic effects, primarily on the basal ganglia, causing numerous behavioral disorders such as anxiety, depression, and memory loss. Chromium, on the other hand, increases the secretion of noradrenaline, a neurotransmitter of the sympathetic nervous system, the activation of which improves cognitive function and memory [20,21].

Bioelements also affect the total (TT) and free (FT) testosterone concentration, as well as estradiol levels, and thus may negatively or positively affect the development of testosterone deficiency syndrome in aging men [22]. 

This study aimed to assess the relationship between the concentration of bioelements and the incidence of depression in men over the age of 50 with a concurrent testosterone deficiency syndrome.

## 2. Material and Methods

### 2.1. Characteristics of the Study Group

This cross-sectional study included 314 men between 50 and 70 years old recruited from primary healthcare facilities (POZ) in Szczecin, Poland. Before the study began, each participant gave their voluntary consent to participate in the study and was acquainted in detail with the course and objectives of the study. The study excluded people undergoing oncological treatment; those with thyroid disease; those receiving neuroleptics, antidepressants, and supplements containing studied bioelements and treated with steroid therapy; those undergoing testosterone replacement therapy; individuals with depression at any stage diagnosed by a psychiatrist; or those diagnosed with TDS.

**Figure 1:**
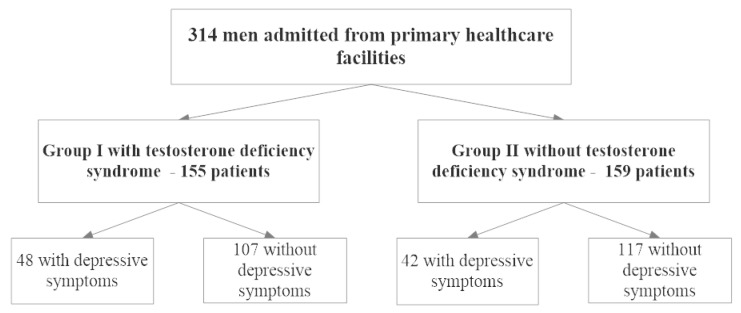
Study flowchart (STROBE). Data regarding education, smoking and marital status, occupational activity, and chronic diseases such as diabetes and hypertension were also collected. Body mass index (BMI) was calculated on the basis of height and weight.

On the basis of the presence of testosterone deficiency syndrome, participants were assigned to one of two groups. Group I included 155 people with TDS. Group II included 159 people without TDS. Patients in both groups were assigned to a subgroup with and without the depressive disorder, which was diagnosed on the basis of the Beck Depression Inventory (BDI-Ia). Research procedures and analyses were the same for all participants. The study flowchart (according to STROBE guidelines) is shown in Figure 1. 

### 2.2. Ethical Considerations

The study was performed in accordance with the Declaration of Helsinki after it was approved by the Bioethical Committee of the Pomeranian Medical University in Szczecin, Poland (2012-12-10 no. KB-0012/159/12). Each patient enrolled in the study signed an informed consent form for participation and study procedures. In terms of personal data protection, the analysis was performed on dehumanized (anonymous) data. 

### 2.3. Study Questionnaires

Three types of questionnaires were used in the study: a dedicated questionnaire prepared by the authors, the Morley questionnaire, and the Beck Depression Inventory (BDI-Ia). The first questionnaire included an interview regarding basic sociodemographic data and medical history information, focusing on determining the presence of possible contraindications in participating in the study. 

The questionnaire created by J.E. Morley was used to assess the symptoms of testosterone deficiency [23]. It consisted of 10 closed questions that were single choice, to which possible “yes” or “no” answers primarily focused on sex drive, physical activity, exercise tolerance, and well-being. This questionnaire, despite being a very good tool for assessing the occurrence of symptoms associated with TDS, does not provide any information on their severity.

The Beck Depression Inventory (BDI-Ia) was used to measure the severity of depressive symptoms and included 21 multiple-choice questions with a range of disjunctive questions related to current mood, level of self-esteem, relationships with other people, appetite, quality of sleep, and fear for one’s health and life [24]. The answers were summarized and compared to the standards outlined in the questionnaire in which a score between 26 and 29 points means high intensity of depressive symptoms, 20–25 points means medium severity, 12–19 points means mild severity, and the result of ≤11 points means no depressive symptoms.

### 2.4. Bioelement Analysis by Spectrophotometry

In patients from both study groups, 9 mL of fasting blood was drawn from a venipuncture into tubes with gel separator and clot activator, which were then centrifuged and stored at −80 °C in 1.5 mL microtubes. Bioelements were analyzed by optical emission spectrometry with inductively coupled plasma (ICP-OES, ICAP 7400 Duo, Thermo Scientific; Waltham, MA, USA). The analysis was carried out in axial and radial orientation. Tested bioelements included magnesium (Mg), manganese (Mn), chromium (Cr), copper (Cu), and zinc (Zn).

Samples of 0.75 ml were thawed to room temperature and microwaved with the MARS 5 system, CEM. Next, the material was transferred to polypropylene tubes, 2 ml of 65% HNO_3_ (Suprapur, Merck; Darmstadt, Germany) was added to each tube, and the tubes were left for 30 min. After this time, 1 ml of unstabilized 30% H_2_O_2_ solution (Suprapur, Merck) was added. Next, all samples were placed in Teflon vessels and heated at 180 °C for 35 min using a microwave system. After this time, the samples were cooled to room temperature and transferred to 15 ml acid-washed polypropylene tubes. A 25-fold dilution was made before ICP-OES measurement. A volume of 400 µL was taken and enriched with a standard to a final concentration of 0.5 mg/L yttrium 1 ml 1% Triton (Triton X-100, Sigma; Darmstadt, Germany). It was then diluted with 0.075% nitric acid (Suprapur, Merck) to give a volume of 10 ml. The samples were stored at the temperature of 8 °C. A blank sample was prepared by adding 300 µL of nitric acid and diluting it in the same way as the test sample.

Multifactor calibration standard samples for Cu, Cr, Mg, Mn, Zn, Se, and Mo analysis and phosphorus ICP Standard (AccuStandard, Inc. New Haven, CT, USA) were prepared with different concentrations of elements, as well as the test and the blank sample. Deionized water (Direct Q UV, Millipore, approximately 18.0 MΩ) was used. The wavelengths (in nanometers) for individual elements were as follows: Cr—283.563 nm, Cu—324.754 nm, Mg—285.213 nm, Mn—260.569 nm, Zn—213.856 nm. The analyses were performed in the Biochemical Laboratory of the Pomeranian Medical University.

### 2.5. Hormone Level Determination and Diagnosis of Testosterone Deficiency Syndrome

Total testosterone (TT), free testosterone (FT), estradiol (E2), and dehydroepiandrosterone sulfate (DHEAS) levels were determined by ELISA, using reagents in ready-made kits (DRG Medtec; Warsaw, Poland) at the Medical Analytics Department of the Laboratory Diagnostics Department of Pomeranian Medical University in Szczecin.

Testosterone deficiency syndrome (TDS) was diagnosed on the basis of the results of laboratory tests with a TT result of less than 2.5 ng/ml or 3.5–2.5 ng/ml when clinical symptoms were assessed using the ADAM (Androgen Deficiency in Aging Male) questionnaire, supplemented by affirmative answers to questions proposed by the European Menopause and Andropause Society referring to the less frequent occurrence of morning erections and sexual thoughts and more frequent erectile dysfunction [25].

### 2.6. Statistical Analysis

The normality of quantitative data distribution was assessed using the Shapiro–Wilk test. Quantitative data were evaluated using the Mann–Whitney U test. Qualitative variables were analyzed using the chi-squared test or chi-squared test with Yates correction. Multivariate logistic regression analysis adjusted for age, smoking and marital status, education, occupational activity, body mass index (BMI), and waist-hip ratio (WHR) classifications was performed. The statistical analysis was performed using the Statistica 13 licensed program (StatSoft, Inc. Tulsa, OK, USA). A *p*-value of ≤0.05 was regarded as statistically significant.

## 3. Results

The full characteristics of the study group are presented in Table 1. Among the respondents (average age of 61.36 ± 6.38 years), 90 men were diagnosed with emotional disturbances manifested as depressive symptoms, which constituted 28.7% of all participants. A total of 155 or 49.3% of participants were diagnosed with testosterone deficiency syndrome.

The results regarding the parameters included in the analysis are presented in Table 2.

Out of participants from the first study group, an increase in mean manganese (*p* = 0.005) and chromium (*p* = 0.022) levels was observed in patients with a known testosterone deficiency syndrome. 

Out of the participants in the second study group, in patients without known testosterone deficiency syndrome, there were no significant changes in the average values of bioelements depending on the presence of depression. The results are presented in Table 3.

The analysis also showed no significant correlation between the selected concentrations of bioelements and depression among patients from the group without known testosterone deficiency syndrome and with TDS. In the TDS group, the analysis showed a significant correlation between manganese (*R* = 0.225, *p* = 0.005) and chromium (*R* = 0.185, *p* = 0.022) levels with the presence of depression. The results are presented in Table 4.

Logistic regression was performed to analyze the data obtained from participants with depressive symptoms and testosterone deficiency syndrome, which was corrected for age, smoking and marital status, education, occupational activity, BMI, and WHR classifications. The results are shown in Table 5. 

## 4. Discussion

Our research hypothesis was to show a relationship between bioelement in patients with depression and co-occurring testosterone deficiency. On the basis of logistic regression, the study showed a significant relationship between the increase in manganese and chromium levels with depressive symptoms and the incidence of testosterone deficiency syndrome in men over 50 years of age. The other bioelements tested such as magnesium, selenium, copper, zinc, and molybdenum did not show significant differences in concentration between the control and the examined patients. A significant correlation between depressiveness and manganese concentrations may confirm its neurotoxic effect on the basal ganglia, resulting in cognitive, emotional, and anxiety disorders, as well as on the induction of oxidative stress causing cell death. These observations have been broadly described in scientific works by, among others, Dukhande et al. in 2006 [26].

In addition, other explanations of the pathomechanism of depressive disorders correlated with an increase in manganese concentrations can also be found in the literature. This is due to its possible effect on the glutamatergic system [27,28,29,30,31,32] and gamma-aminobutyric acid (GABA) receptors [33]. There are already works suggesting a negative correlation between manganese concentrations and attenuation of cognitive functions observed among children between 6 and 13 years of age [34]. Our research shows that similar changes can also be seen in another age group—aging men. A similar negative correlation of manganese can also be indicated for the TT concentration, which corresponds to the results of our work [22].

A negative correlation between chromium concentration and the incidence of depressive symptoms was observed in studies conducted by Młyniec et al. in 2014. This can be explained by the effect of this bioelement on the regulation of emotional functions and the ability to remember by regulating neurotransmitters and neuromodulators belonging to the monoaminergic system [18,19]. However, due to the small number of studies regarding the relationship between depressive and cognitive disorders and chromium, and given the results presented in this paper, we consider it important to continue research on this bioelement on a larger and more diverse study group. Future studies should also include patients undergoing major surgery or treated in the intensive care unit to evaluate the role of chromium levels in postoperative cognitive disorders and postoperative delirium [35,36].

In the case of testosterone deficiency syndrome, however, we observed a positive correlation between chromium and free testosterone (FT) levels and negative fractions in the case of sex hormone binding globulin (SHBG), suggesting a positive effect of this bioelement on patients suffering from TDS [22]. In the research by Serefko et al. in 2016, the positive effect of magnesium was described for a number of biochemical pathways in the brain, the GABAergic system, as well as the monoaminergic system, which may lead to a reduction of symptoms in patients suffering from personality disorders, anxiety disorders, and even clinical depression [17]. Rajizadeh et al. in research performed in 2017 examined 60 people suffering from magnesium deficiency and symptomatic depression, demonstrating that taking 500 mg of magnesium for more than 8 weeks has positive results in the treatment of both these disorders and diseases [37].

On the other hand, a proven positive correlation between magnesium concentration and TT concentration may show the significance of this bioelement when determining the pathomechanisms of testosterone deficiency syndrome [38]. In our work, however, we did not find any relationship between depression and magnesium levels in either people with testosterone deficiency syndrome or those without it.

A meta-analysis carried out by Ni et al. in 2018, with results collected from other studies, indicated a significant relationship, one that increases with age, between the incidence of depression symptoms and copper concentration. The authors additionally suggested that this bioelement is a biomarker for a full-blown disease [39]. This was confirmed when analyzing the effect of this bioelement on the glutamatergic, GABAergic and monoaminergic systems [18,19]. However, due to the insufficient amount of research conducted on these relationships, the role of copper in the mechanisms of depressive disorders still remains controversial. Studies by Chang et al. in 2011 showed a negative correlation between serum testosterone level and copper concentrations [40]. However, in our research, no significant relationship was observed between copper and the concentrations of male steroid hormone and depressiveness in aging men.

The relationship between depressive and anxiety disorders and zinc is one of the most extensively described in the literature around issues related to this work [41,42]. In animal studies, supplementation with this bioelement was shown to even have a positive effect on the treatment of clinical depression [18]. It may be related to the zinc involvement in the function of many systems described earlier that regulate cognitive, emotional, and memory functions, i.e., the glutamatergic, GABAergic, and monoaminergic systems [18,19]. Additionally, this element has been shown to modulate the 5-HT receptors (in particular type 5-HT_1A_), as is the case with SSRIs, which reduce receptor sensitivity and inhibit serotonin reuptake in the synaptic cleft. This mechanism has been broadly described by Duboszewska et al. [43]. Moreover, zinc deficiency affects the incidence of inflammatory processes by influencing interleukin (IL)-1β production. This element also helps to reduce oxidative stress that leads to neurodegeneration and cell death in the nervous system [42,44]. 

Additionally, in the case of testosterone deficiency syndrome, zinc deficiency seems to be one of the components of the pathomechanism due to the negative correlation with SHBG, causing the body’s access to free testosterone fractions [22]. Other studies have shown that zinc is inversely correlated with glycemic levels in type II diabetes [45]. In 2007, Spark et al. described the relationship between type II diabetes and a decrease in testosterone levels [46]. Studies by Kelishadi et al. from 2010 proved that zinc deficiency affects an increase in plasma total and low-density lipoprotein (LDL) cholesterol and an increase of BMI [47]. Increases in body fat tissue leading to obesity are also a risk factor for testosterone deficiency syndrome. It is associated with the aromatase enzyme present in this tissue, which is responsible for an irreversible transformation of testosterone into estradiol [48]. However, our research has not confirmed the relationship between zinc and testosterone deficiency syndrome, which may be due to the small study group.

## 5. Conclusions

The results of our study showed the relationship between manganese and chromium concentration and the occurrence of depression in men over 50 years of age with a concurrent testosterone deficiency syndrome. This may indicate the relationship between these bioelements, and emotional disorders manifested as depressive symptoms in aging men with a diagnosed testosterone deficiency.

### Limitations

The most significant limitation of our study was the ambiguity of the classification criteria of testosterone deficiency syndrome. There are many standards set by various TDS testing organizations around the world. However, in the above study, we chose those accepted by the International Society of Andrology (ISA), International Society for the Study of the Aging Male (ISSAM), European Association of Urology (EAU), European Academy of Andrology (EAA), and American Society of Andrology (ASA) in 2009. An additional difficulty is the limited amount of literature available on bioelements and their impact on the functioning of cognitive processes. Moreover, the power of the investigations was obtained slightly lower than 0.8; therefore, our results need confirmation in a larger patient population with depressive symptoms and TDS. No information related to alcohol consumption, detailed medical history, or physical activity that could affect the results presented in the study was collected. We believe that in order to increase the credibility of the results, the number of study participants should be increased in the future. Free testosterone determination was performed by ELISA, which may cause inaccuracy in the determination of this parameter.

## Figures and Tables

**Table 1:** Study group characteristics.

**Demographic Data**		
Age (years), (mean ± SD; Me)		61.36 ± 6.37; 62
BMI (kg/m^2^), (mean ± SD; Me)		28.27 ± 4.37; 27.73
BMI classification	norm	63 (20.06%)
	overweight	164 (52.23%)
	first degree obesity	65 (20.70%)
	second degree obesity	15 (4.78%
	third degree obesity	7 (2.23%)
Hips (cm), (mean ± SD; Me)		103.26 ± 7.71; 103
Waist (cm), (mean ± SD; Me)		102.07 ± 12.04; 100
WHR classification	norm	208 (66.24%)
	abdominal obesity	106 (33.76%)
Education (*n*, %)	primary	17 (5.42%)
	vocational	54 (17.20%
	secondary	128 (40.76%)
	higher	115 (36.62%)
Smoking (*n*, %)	non-smoker	263 (83.76%)
	smoker	51 (16.24%)
Occupational activity (*n*, %)	employed	19 (6.05%)
	pensioner (due to health condition)	137 (43.63%)
	pensioner (due to age)	37 (11.78%)
	unemployed	121 (38.54%)
Marital status (*n*, %)	married	48 (15.29%)
	unmarried	266 (84.71%)
Statin treatment (*n*, %)		50 (15.92%)
Diabetes (*n*, %)		54 (17.20%)
Hypertension (*n*, %)		172 (54.78%)
Depressive symptoms (*n*, %)		90 (28.66%)
Total testosterone deficiency (*n*, %)		155 (49.36%)
**Serum Bioelements**		
Mn (mg/L), (mean ± SD; Me)		0.002 ± 0.001; 0.002
Zn (mg/L) (mean ± SD; Me)		0.889 ± 0.131; 0.880
Cu (mg/L) (mean ± SD; Me)		1.083 ± 0.179; 1.065
Cr (mg/L) (mean ± SD; Me)		0.0005 ± 0.0002; 0.0004
Mg (mg/L) (mean ± SD; Me)		0.002 ± 0.001; 0.002

**Legend:** SD—standard deviation, Me—median, BMI—body mass index, TAG—triacylglycerol, Mn—manganese, Zn—zinc, Cu—copper, Cr—chromium, Mg—magnesium.

**Table 2:** Tested parameters for group I with a known testosterone deficiency syndrome.

Serum Bioelements	Group I—TDS (*n* = 155)						*p*-Value
	No Depressive Symptoms (*n* = 107)			Depressive Symptoms (*n* = 48)			
	Mean	Median	±SD	Mean	Median	±SD	
Mn (mg/L) *10^−^^3^	1.822	1.630	1.203	2.181	1.970	1.055	0.005
Zn (mg/L)	0.895	0.885	0.144	0.873	0.869	0.119	0.649
Cu (mg/L)	1.078	1.050	0.175	1.116	1.098	0.204	0.186
Cr (mg/L) *10^−^^3^	0.459	0.398	0.267	0.504	0.481	0.191	0.022
Mg (mg/L)	20.697	20.550	2.323	20.389	20.550	2.407	0.782

**Legend**: Mn—manganese, Zn—zinc, Cu—copper, Cr—chromium, Mg—magnesium, SD—standard deviation. *10^−3^ increase to ten to the minus third power.

**Table 3:** Tested parameters for group II without testosterone deficiency syndrome.

Serum Bioelements	Group II—No TDS (*n* = 159)						*p*-Value
	No Depressive Symptoms (*n* = 117)			Depressive Symptoms (*n* = 42)			
	Mean	Median	±SD	Mean	Median	±SD	
Mn (mg/L) *10^−^^3^	1.723	1.580	1.010	1.866	1.515	1.327	0.874
Zn (mg/L)	0.893	0.880	0.128	0.878	0.904	0.119	0.980
Cu (mg/L)	1.082	1.070	0.161	1.058	1.055	0.213	0.629
Cr (mg/L) *10^−^^3^	0.466	0.446	0.178	0.439	0.399	0.197	0.240
Mg (mg/L)	21.338	21.280	1.801	20.876	20.985	2.680	0.095

**Legend:** Mn—manganese, Zn—zinc, Cu—copper, Cr—chromium, Mg—magnesium, SD—standard deviation. *10^−3^ increase to ten to the minus third power.

**Table 4:** Correlation between the concentration of bioelements and depressive symptoms in patients with and without testosterone deficiency syndrome.

Serum Bioelements	Group I—TDS (*n* = 155)		Group II—No TDS (*n* = 159)	
	Depressive Symptoms (*n* = 48)		Depressive Symptoms (*n* = 42)	
	*R*	*p*-Value	*R*	*p*-Value
Mn (mg/L)	0.225	0.005	−0.013	0.873
Zn (mg/L)	−0.037	0.649	−0.002	0.978
Cu (mg/L)	0.107	0.187	−0.039	0.630
Cr (mg/L)	0.185	0.022	−0.094	0.240
Mg (mg/L)	−0.022	0.782	−0.133	0.095

**Legend:** Mn—manganese, Zn—zinc, Cu—copper, Cr—chromium, Mg—magnesium.

**Table 5:** Multivariate regression analysis for patients with depressive symptoms and with testosterone deficiency syndrome.

	Odds Ratio	95% CI	*p*-Value
Mn (mg/L)	1.394	1.008–1.928	0.045
Zn (mg/L)	0.999	0.996–1.002	0.473
Cu (mg/L)	1.001	0.999–1.004	0.206
Cr (mg/L)	3.531	0.791–15.766	0.098
Mg (mg/L)	1.000	1.000–1.000	0.452

**Legend:** CI—confidence interval, Mn—manganese, Zn—zinc, Cu—copper, Cr—chromium, Mg—magnesium.
